# Supplementary figures and images for: Comprehensive analysis of transcriptomics and radiomics revealed the potential of TEDC2 as a diagnostic marker for lung adenocarcinoma
Source: PeerJ. 2024 Nov 14;12:e18310. doi: 10.7717/peerj.18310 (PMC11569783; doi:10.7717/peerj.18310)

**A****Scale independence**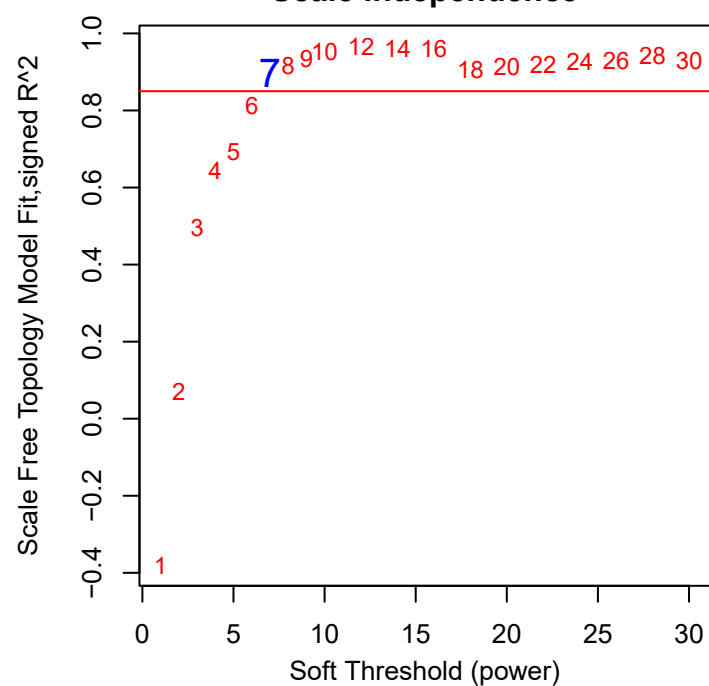**B****Mean connectivity**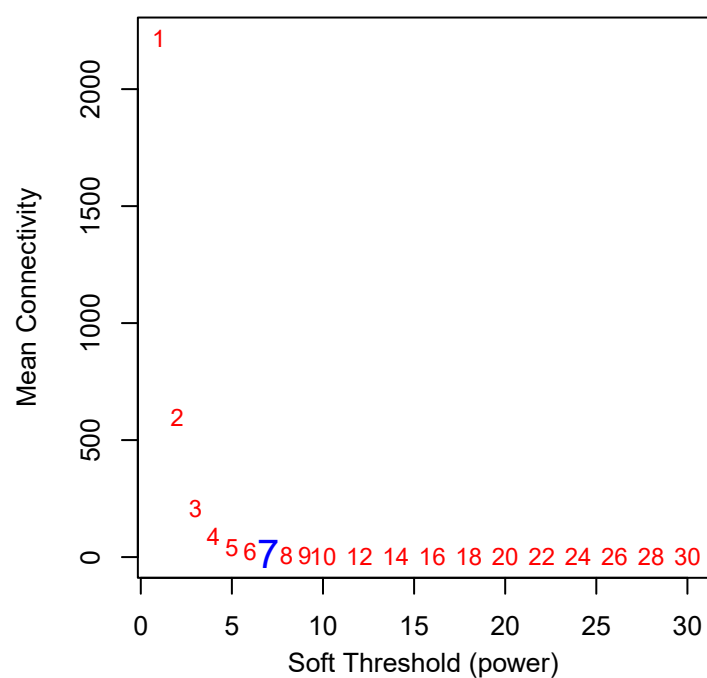**C**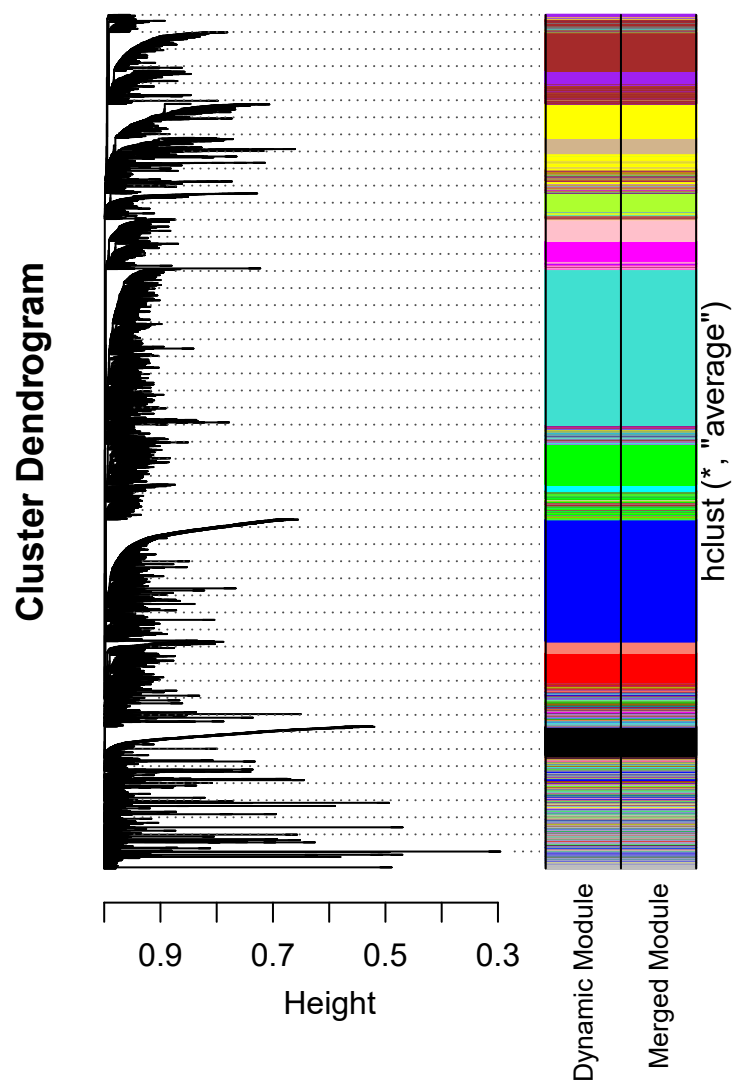**D**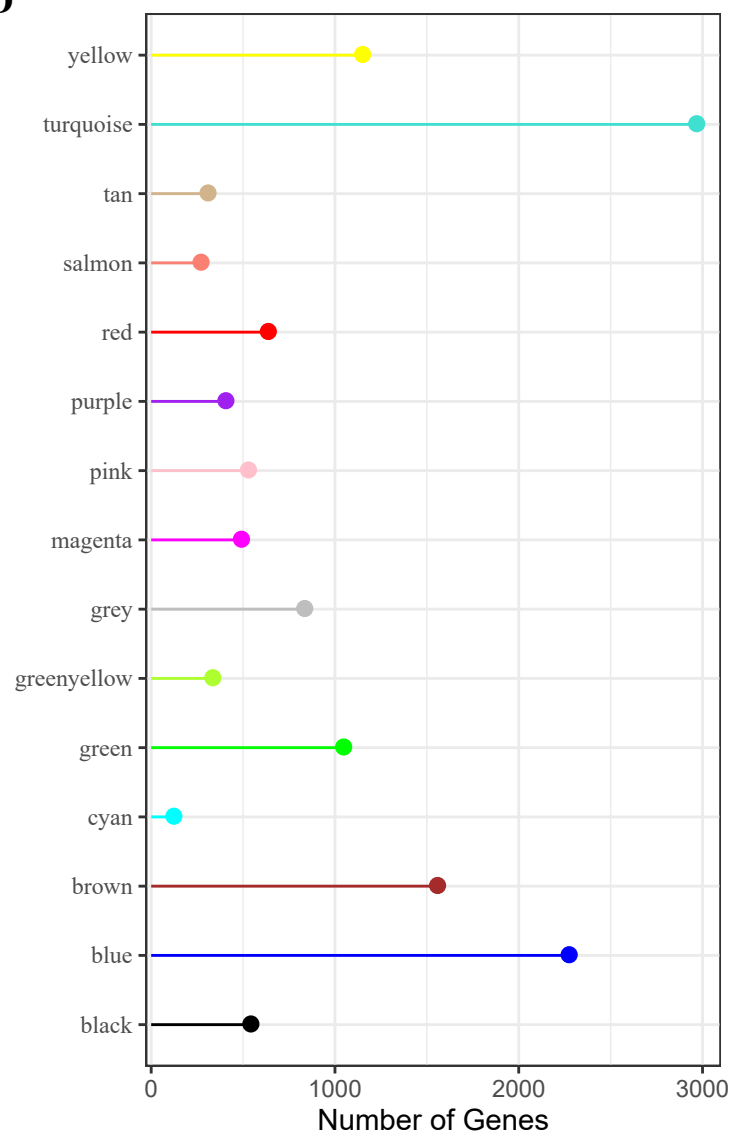

Supplement: Supplemental Information 1 — The relationship of soft threshold with scale free topology (A) and mean connectivity (B). (C) Hierarchical clustering tree obtained by WGCNA. (D) Number of genes with similar expression patterns clustered in each module. [file peerj-12-18310-s001.pdf]

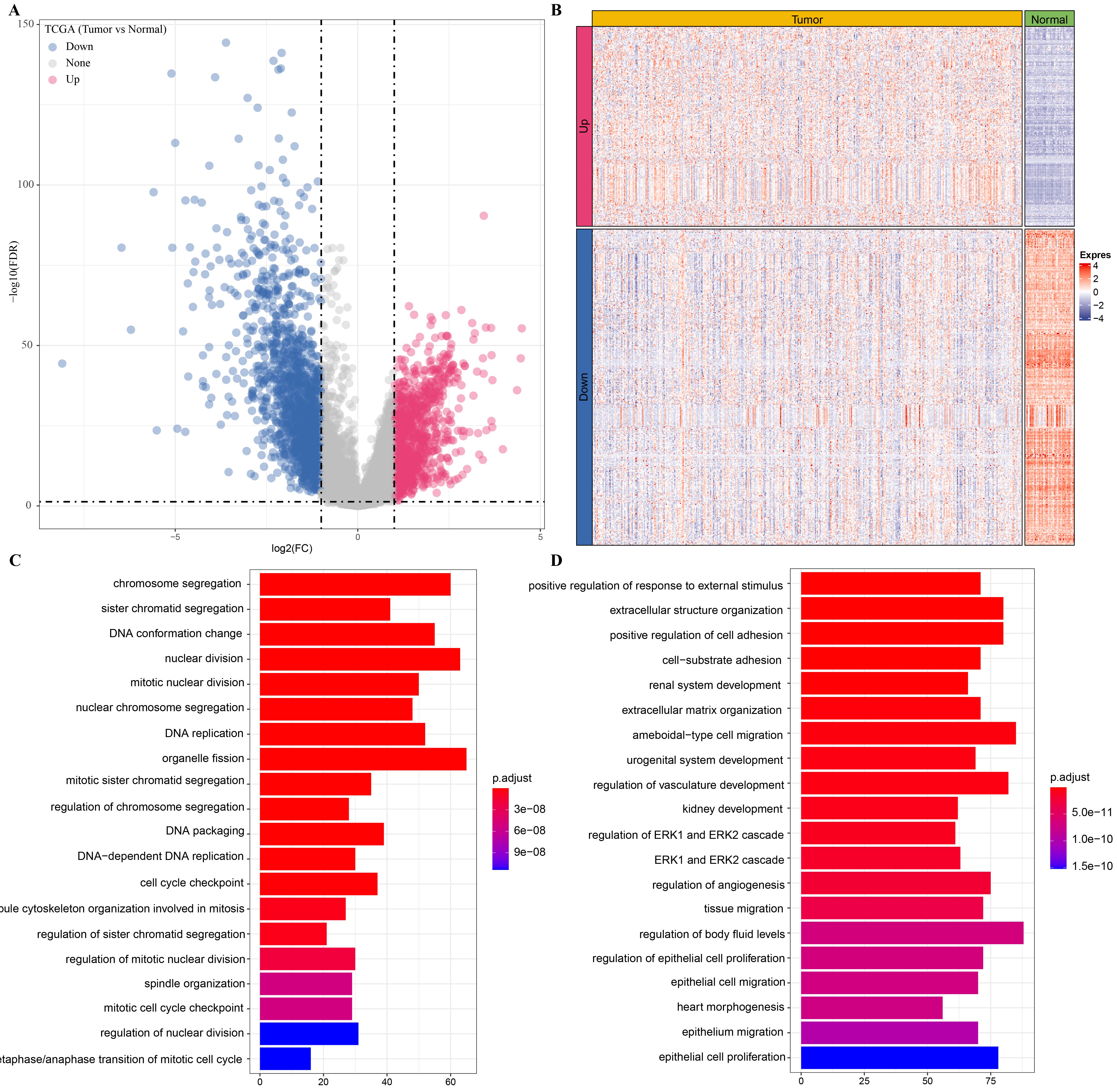

Supplement: Supplemental Information 2 — (A) DEGs in LUAD samples compared to normal samples, the blue points are down-regulated DEGs and the red points are up-regulated DEGs in LUAD samples. (B) Heatmap shows the expression of DEGs between LUAD and normal samples. (C) The 20 pathways significantly enriched by up-regulated DEGs in LUAD were ranked from small to large according to P value. (D) The 20 pathways significantly enriched by down-regulated DEGs in LUAD were ranked from small to large according to P value. [file peerj-12-18310-s002.jpg]

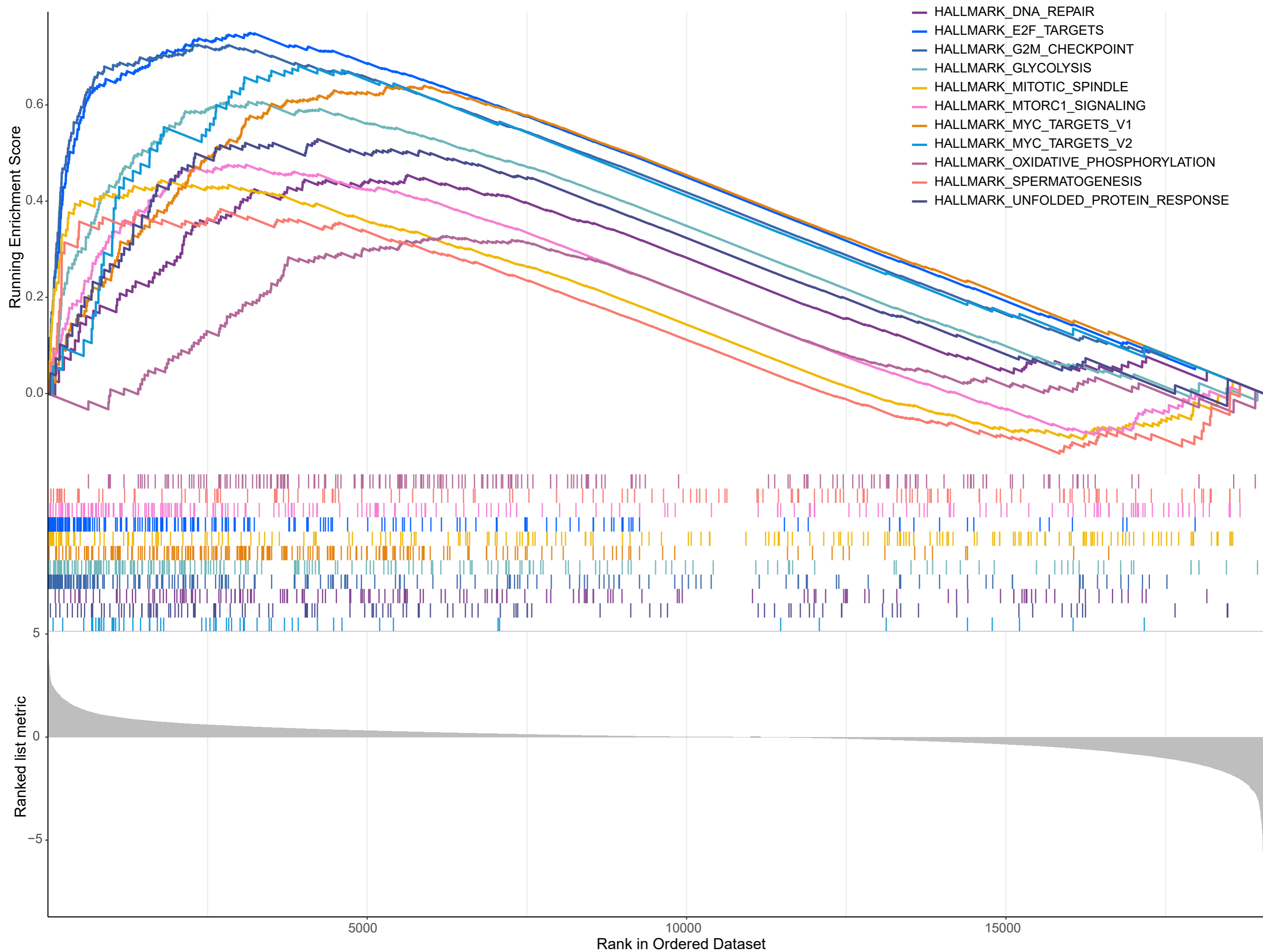

Supplement: Supplemental Information 3 [file peerj-12-18310-s003.pdf]
