# Supplementary material for: Comprehensive analysis of transcriptomics and radiomics revealed the potential of TEDC2 as a diagnostic marker for lung adenocarcinoma
Source: PeerJ. 2024 Nov 14;12:e18310. doi: 10.7717/peerj.18310 (PMC11569783; doi:10.7717/peerj.18310)
Supplement: Supplemental Information 4 — Univariate Cox regression analysis for diagnostic markers as well as clinical variables with (A) OS and (B) PFI in patients. Multivariate Cox regression forest plot for (C) OS and (D) PFI of LUAD patients. [file peerj-12-18310-s004.pdf]

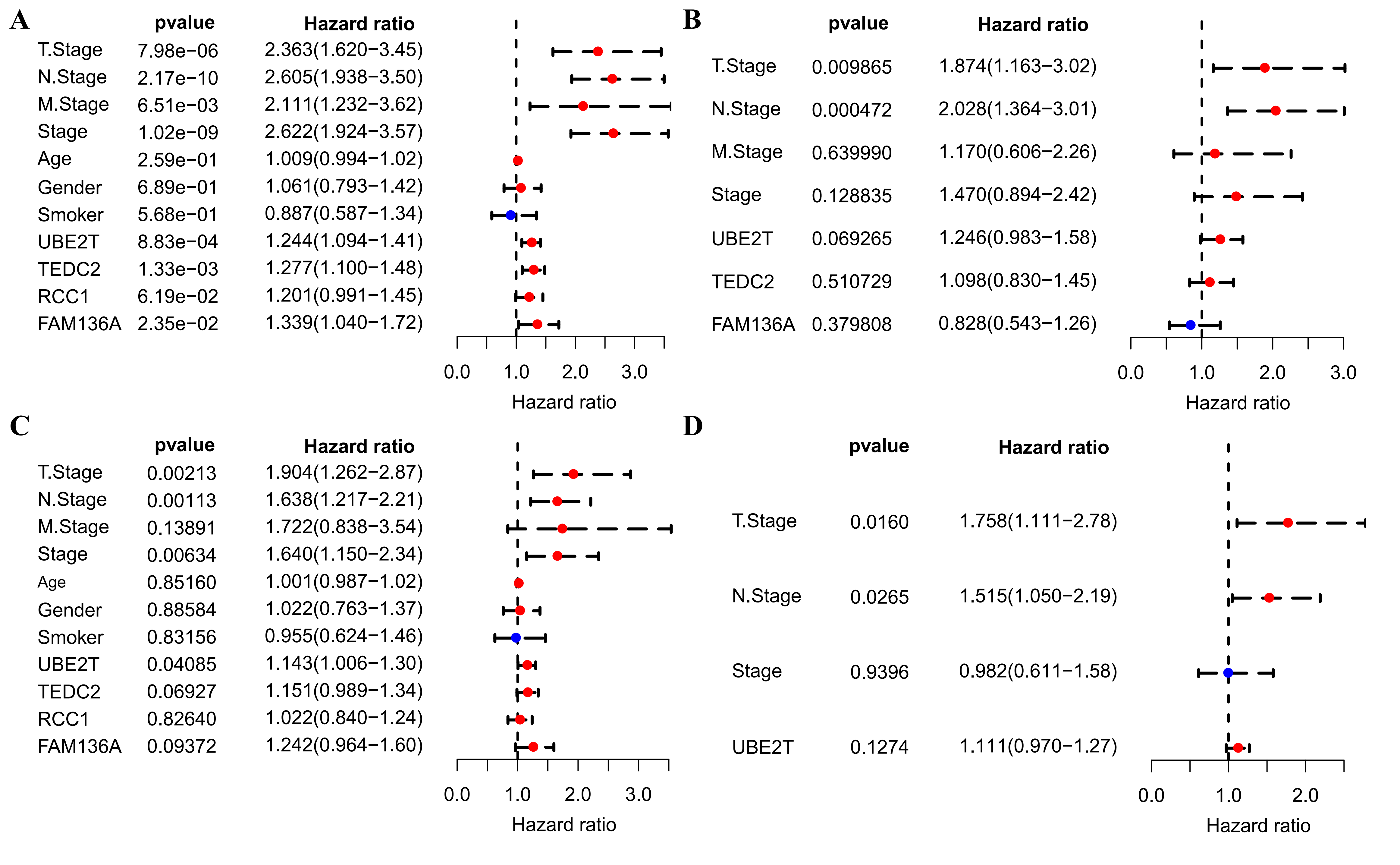
A
pvalue
Hazard ratio
T.Stage 7.98e−06 2.363(1.620−3.45)
N.Stage 2.17e−10 2.605(1.938−3.50)
M.Stage 6.51e−03 2.111(1.232−3.62)
Stage 1.02e−09 2.622(1.924−3.57)
Age 2.59e−01 1.009(0.994−1.02)
Gender 6.89e−01 1.061(0.793−1.42)
Smoker 5.68e−01 0.887(0.587−1.34)
UBE2T 8.83e−04 1.244(1.094−1.41)
TEDC2 1.33e−03 1.277(1.100−1.48)
RCC1 6.19e−02 1.201(0.991−1.45)
FAM136A 2.35e−02 1.339(1.040−1.72)
0.0 1.0 2.0 3.0
Hazard ratio
B
pvalue
Hazard ratio
T.Stage 0.009865 1.874(1.163−3.02)
N.Stage 0.000472 2.028(1.364−3.01)
M.Stage 0.639990 1.170(0.606−2.26)
Stage 0.128835 1.470(0.894−2.42)
UBE2T 0.069265 1.246(0.983−1.58)
TEDC2 0.510729 1.098(0.830−1.45)
FAM136A 0.379808 0.828(0.543−1.26)
0.0 1.0 2.0 3.0
Hazard ratio
C
pvalue
Hazard ratio
T.Stage 0.00213 1.904(1.262−2.87)
N.Stage 0.00113 1.638(1.217−2.21)
M.Stage 0.13891 1.722(0.838−3.54)
Stage 0.00634 1.640(1.150−2.34)
Age 0.85160 1.001(0.987−1.02)
Gender 0.88584 1.022(0.763−1.37)
Smoker 0.83156 0.955(0.624−1.46)
UBE2T 0.04085 1.143(1.006−1.30)
TEDC2 0.06927 1.151(0.989−1.34)
RCC1 0.82640 1.022(0.840−1.24)
FAM136A 0.09372 1.242(0.964−1.60)
0.0 1.0 2.0 3.0
Hazard ratio
D
pvalue
Hazard ratio
T.Stage 0.0160 1.758(1.111−2.78)
N.Stage 0.0265 1.515(1.050−2.19)
Stage 0.9396 0.982(0.611−1.58)
UBE2T 0.1274 1.111(0.970−1.27)
0.0 1.0 2.0
Hazard ratio
